# Jaborandi in Cholera Infantum and Scarlatina

**Published:** 1876-07

**Authors:** 


*Jaborandi in Cholera Infantum and Scarlatina.*—Dr. Wilson has used jaborandi in one case of cholera infantum, in order to determine the liquids from the intestines to the skin, and its administration was followed by the happiest effect. The Doctor is also using jaborandi in a case of scarlatina, with admirable effects.—*Richmond and Louisville Med. Jour.*

